# Barriers to a healthy lifestyle among patients attending primary care clinics at a university hospital in Riyadh

**DOI:** 10.4103/0256-4947.51818

**Published:** 2009

**Authors:** Aljoharah M. AlQuaiz, Salwa A. Tayel

**Affiliations:** From the Department of Family and Community Medicine, College of Medicine, King Saud University, Riyadh, Saudi Arabia

## Abstract

**BACKGROUND AND OBJECTIVES:**

The occurrence and progress of chronic non-communicable diseases (NCDs) is associated with unhealthy lifestyles and behaviors. Modification of barriers to healthy lifestyle can produce great benefits. The objective of this study was to identify barriers to physical activity and healthy eating among patients attending primary health care clinics in Riyadh city.

**PATIENTS AND METHODS:**

A cross-sectional study was conducted at King Khalid University Hospital (KKUH) in Riyadh city. Four hundred and fifty participants attending primary health care clinics (PHCC) from 1 March to 30 April 2007 were randomly selected. A questionnaire about barriers to physical activity and healthy eating was adapted from the CDC web site.

**RESULTS:**

The prevalence of physical inactivity among the Saudi population in the study was 82.4% (371/450). Females were more physically inactive (87.6%, 268/306) compared to males (71.5%, 103/144) (*P*<.001). The most common barrier to physical activity was lack of resources (80.5%, 326/405), which was significantly higher among females than males and among the lower income versus the higher income group. The most common barrier to healthy diet was lack of willpower. More than four-fifths (80.3%, 354/441) of the study group stated that they did not have enough will to stick to a diet.

**CONCLUSION:**

Lack of resources was the most important barrier for physical activity, while lack of willpower and social support were both barriers for adherence to physical activity and a healthy diet.

The alarming burden of chronic non-communicable diseases (NCDs) is of great concern to public health in both developed and developing countries. Currently, 52% of the Eastern Mediterranean region's disease burden is due to NCDs and this burden is expected to rise to 60% by 2020.^1^ The occurrence and progress of NCDs is associated with unhealthy lifestyles and behaviors.^1^ Sedentary lifestyles increase all causes of mortality, double the risk of cardiovascular diseases, diabetes, and obesity and increase the risks of colon cancer, high blood pressure, osteoporosis, lipid disorders, depression and anxiety. Physical activity and proper nutrition are critical for improving health and extending life.^2^

It is promising that much of the global burden of chronic NCDs is preventable through regular physical activity and good nutrition.^3^ However, the trend in global physical inactivity is alarming. Despite all the advantages of exercise, at least 60% of the global population are inactive.^4^ The prevalence of physical inactivity in Saudi Arabia is reported to be high.^5^,^6^ This is coupled with the high prevalence of chronic diseases in the kingdom compared to other parts in the world.^6^ In addition, a large gap remains between healthy dietary patterns and what people actually eat. Research has demonstrated that lifestyle change is related to a variety of factors, including social, psychological, cultural and environmental factors.^7^ Great benefits and hopefully modification of these barriers can be acquired only if these barriers are well known and apprehended.^8^

There is limited published research about barriers to healthy lifestyle, especially in developing countries. Research is essential to propose an appropriately tailored and culturally relevant approach for intervention. The objective of this study was to identify barriers to physical activity and healthy eating among patients attending primary health care clinics in Riyadh city.

## PATIENTS AND METHODS

A cross-sectional study was conducted at King Khalid University Hospital (KKUH) in Riyadh city. Participants attending primary health care clinics (PCC) from 1 March to 30 April 2007 were randomly selected using a simple random technique. Patients were invited to participate in the study on three randomly selected days per week. All cooperative participants were included. The sample size was calculated using an estimated 96% prevalence of physical inactivity as reported by previous studies in the Saudi population.^6^ For 95% confidence limits and a 2% error in the prevalence rate, the calculated sample size was 370 participants. Anticipating a response rate of 70% to 80%, the total sample size was 450 subjects using Epi-info (Centers for Disease Control, Atlanta, Georgia, USA; *http://www.cdc.gov/epiinfo/*) Inclusion criteria were Saudi nationality and age ≥15 years. Participants who had difficulty in walking were excluded.

Data were collected through self-administered questionnaire. Those who were illiterate or needed clarification were interviewed. The questionnaire included questions about sociodemographic variables (age, marital status, occupation, education and economic status) and level of physical activity. Questions about barriers to physical activity and healthy diet were adapted from a questionnaire from the CDC web site.^9^ The questionnaire included 21 questions for barriers to physical activity. A scoring system was used to indicate how likely a person would answer each statement about barriers (very likely=3, somewhat likely=2, somewhat unlikely=1, very unlikely=0). Scores of three related questions were added to identify a category of barriers. The maximum possible score of one category was 9. A score of 5/9 or above in any category would indicate an important barrier. A group of three related questions were used to identify one category as a barrier to physical activity. Barriers were categorized into one of seven categories; lack of time, lack of social influence, lack of energy, lack of resources, lack of willpower, fear of injury and lack of skill. Furthermore, the questionnaire included 15 questions for barriers to healthy diet. A group of three related questions determined one category as a barrier to a healthy diet. A similar scoring system was used for subjects answering statements about barriers. Thus, barriers to healthy food were categorized into one of five categories: lack of willpower, lack of knowledge, lack of time, low socioeconomic state and lack of social influence. The questionnaire was translated to Arabic (see at end of article on *www.saudiannals.net),* pre-tested and validated by distributing 30 formats randomly to participants while waiting for their medical appointments at PCC. Modifications were made as necessary.

According to Recommendations of American College of Sports Medicine and the Centers for Disease Control and Prevention (ACSM/CDC), regular physical activity was defined when a person practices moderate-intensity sport and leisure time physical activity regularly for at least 30 minutes on five or more days per week. Irregular physical activity was defined as not achieving the recommended amount of regular sport and leisure time physical activity. Inactive was defined as not practicing any regular sport and leisure time physical activity. Healthy diet was defined as a diet meeting the American Heart Association's recommendations for 2006 at 2000 calories/day.10 Data entry and analysis was done using the Statistical Package for the Social Sciences (SPSS version 11). Descriptive statistics and the chi-square test were used for analysis. The level of significance was set at *P*<.05.

## RESULTS

The ages of the 450 participants ranged from 15 to 80 years. Nearly half were in the age group 15 to 29 years. Their mean (SD) age was 33.28 (13.3) years. Nearly one-third of the subjects were males (32.0%). Less than one-third (29.4%) were housewives. About half (48.4%) had a university or higher education. Two-fifths (40.4%) were never married and about two-thirds had incomes less than 10 000 SR per month. None of the participants practiced regular physical activity at the recommended levels of the CDC/ACSM (Table 1). Only 17.6% of the study group practiced irregular physical activity for 15 minutes or more three times per week. Thus the prevalence of physical inactivity among Saudis in our study was 82.4%. Females were more physically inactive (87.6%) compared to males (71.5%). Similarly, physical inactivity was significantly higher among younger age groups.

**Table 1:** Sociodemographic characteristics by level of physical activity.

Sociodemographic characteristic	Irregular physical activity		Physically inactive	
	No.	%	No.	%
**Total (n=450)**	**79**	**17.6**	**371**	**82.4**

Gender				
Males (n=144)	41	28.5	103	71.5
Females (n=306)	38	12.4	268	87.6

	χ^2^=17.43, ***P*<.001**			

Age				
15-29 years (n=214)	34	15.9	180	84.1
30-45 years (n=110)	14	12.7	96	87.2
>45 years (n=100)	26	26.0	74	74.0

	χ^2^=7.25, ***P*=.027**				

Education				
Below university (n=231)	45	19.5	186	80.5
University and higher (n=217)	34	15.7	183	84.3

	χ^2^=1.12, *P*=.29				

Marital status				
Ever married (n=264)	50	18.9	214	81.1
Never married (n=179)	29	16.2	150	83.8

	χ^2^=0.546, *P*=.46				

Income per month				
<10 000SR (n=279)	53	19.0	226	81.0
>10 000SR (n=156)	26	16.7	130	83.3

	χ^2^=0.365, *P*=.546

Totals do not always equal total number of patients because of missing data. Bolding indicates statistically significant difference.

The most likely reason for being inactive was lack of resources (80.5%) (Table 2), which was significantly higher in females than males and in the lower versus higher income group. Lack of willpower ranked second as a barrier to exercise. More than three-quarters of the study group (76.8%) had been thinking about getting more exercise. Lack of social support was reported by 76.8% of study group to be a barrier to physical activity. This was significantly higher among females than male subjects. Lack of energy was stated by about three-quarters of study group as a barrier to exercise (73.2%). Lack of energy was significantly higher among females than male subjects.

**Table 2:** Sociodemographic characteristics by barriers to physical activity.

Sociodemographic characteristic	Lack of resources		Lack of willpower		Lack of social support		Lack of energy		Lack of skills		Lack of time		Fear of injury	
	No.	%	No.	%	No.	%	No.	%	No.	%	No.	%	No.	%
**Total (n=450)**	**326/405**	**80.5**	**311/405**	**76.8**	**315/410**	**76.8**	**298/407**	**73.2**	**178/409**	**43.5**	**174/425**	**40.9**	**86/406**	**21.2**

Gender														
Males	91/126	72.2	91/126	72.2	82/128	64.1	84/129	65.1	40/127	31.5	52/133	39.1	22/126	17.5
Females	235/279	82.2	220/279	78.9	233/282	82.6	214/278	77.0	138/282	48.9	122/292	41.8	64/280	22.9
	χ^2^=7.970	***P*=.005**	χ^2^=2.141	*P*=.143	χ^2^=17.039	***P*<.001**	χ^2^=6.323*	***P*=.012**	χ^2^=10.835	***P*=.001**	χ^2^=0.272	*P*=.602	χ^2^=1.516	*P*=.218

Age														
15-<30 years	161/202	79.7	155/200	77.5	159/207	76.8	148/202	73.3	82/204	40.2	84/210	40.0	23/205	11.2
30-45 years	78/98	79.6	83/101	82.2	81/100	81.0	75/101	74.3	43/99	43.4	48/104	46.2	27/99	27.3
>45 years	70/83	84.3	59/81	72.8	59/79	74.7	59/81	72.8	45/83	54.2	35/87	40.2	32/79	40.5
	χ^2^=0.910	*P*=.634	χ^2^=2.281	*P*=.320	χ^2^=1.116	*P*=.572	χ^2^=0.053	*P*=.974	χ^2^=4.726	*P*=.094	χ^2^=1.176	*P*=.556	χ^2^=31.796	***P*<.001**

Education														
Less than university	162/193	83.9	154/195	79.0	151/197	76.6	142/195	72.8	105/195	53.8	84/210	40.0	65/193	33.7
University and higher	164/210	78.1	156/208	75.0	163/211	77.3	155/210	73.8	72/212	34.0	90/213	42.3	19/211	9.0
	χ^2^=2.221	*P*=.136	χ^2^=0.896	*P*=.344	χ^2^=0.021	*P*=.885	χ^2^=0.051	*P*=.822	χ^2^=16.341	***P*<.001**	χ^2^=0.222	*P*=.638	χ^2^=37.262	***P*<.001**

Marital status														
Ever married	186/230	80.9	183/232	78.9	180/232	77.6	171/230	74.3	109/231	47.2	108/242	44.6	63/229	27.5
Never married	136/168	81.0	122/166	73.5	129171	75.4	121/170	71.2	67/172	39.0	64/176	36.4	20/170	11.8
	χ^2^=0.000	*P*=.983	χ^2^=1.567	*P*=.211	χ^2^=0.254	*P*=.614	χ^2^=0.499	*P*=.480	χ^2^=2.716	*P*=.099	χ^2^=2.874	*P*=.090	χ^2^=14.684	***P*<.001**

Income per month														
<10000SR	207/242	85.5	187/243	77.0	185/244	75.8	176/242	72.7	123/244	50.4	98/258	38.0	61/243	25.1
≥10000SR	111/149	74.5	115/149	77.2	120/151	79.5	114/151	75.5	50/151	33.1	72/152	47.4	23/149	15.4
	χ^2^=7.403	***P***=.007	χ^2^=0.003	*P*=.959	χ^2^=0.707	*P*=.401	χ^2^=0.369	*P*=.544	χ^2^=11.338	***P***=.001	χ^2^=3.470	*P*=.062	χ^2^=5.126	***P***=.024

Bolding indicates statistically significant difference.

The most common barrier to healthy diet was lack of willpower (Table 3). More than four-fifths (80.3%) of the study group stated that they had insufficient will to stick to a diet. Lack of willpower was significantly higher among the middle-aged group (30-45 years) and among those ever married. Lack of social support was reported by about three-quarters (72.4%) of study group to be a barrier to a healthy diet. It was significantly higher among the middle-aged group (30-45 years) and in those with less than a university level of education.

**Table 3:** Sociodemographic characteristics by barriers to healthy diet.

Sociodemographic characteristic	Lack of willpower		Lack of social support		Lack of time		Lack of resources		Lack of knowledge	
	No.	%	No.	%	No.	%	No.	%	No.	%
**Total (n=450)**	**354/441**	**80.3**	**323/446**	**72.4**	**298/441**	**67.6**	**268/445**	**60.2**	**202/436**	**46.3**

Gender										
Males	106/140	75.7	97/140	69.3	90/141	63.8	85/141	60.3	71/136	52.2
Females	248/301	82.4	226/306	73.9	208/300	69.3	183/304	60.2	131/300	43.7
	χ^2^=2.691	*P*=.101	χ^2^=1.005	*P*=.316	χ^2^=1.326	*P*=.250	χ^2^=0.00	*P*=.986	χ^2^=2.744	*P*=.098

Age										
15-<30 years	161/211	76.3	151/213	70.9	159/210	75.7	152/212	71.7	98/209	46.9
30-45 years	95/107	88.8	91/111	82.0	75/110	68.2	60/109	55.0	48/109	44.0
>45 years	78/99	78.8	65/97	67.0	48/98	49.0	43/99	43.4	46/94	48.9
	χ^2^=7.077	***P*=.029**	χ^2^=6.776	***P*=.034**	χ^2^=21.793	***P*<.001**	χ^2^=24.585	***P*<.001**	χ^2^=0.501	*P*=.778

Education										
Less than university	187/226	82.7	177/228	77.6	147/225	65.3	139/227	61.2	116/222	52.3
University and higher	165/213	77.5	144/216	66.7	150/214	70.1	128/216	59.3	85/212	40.1
	χ^2^=1.923	*P*=.166	χ^2^=6.658	***P*=.010**	χ^2^=1.136	*P*=.287	χ^2^=0.180	*P*=.671	χ^2^=6.447	***P*=.011**

Marital status										
Ever married	218/260	83.8	191/261	73.2	158/261	60.5	136/260	52.3	119/252	47.2
Never married	129/174	74.1	125/178	70.2	135/173	78.0	128/178	71.9	80/177	45.2
	χ^2^=6.130	***P*=.013**	χ^2^=0.458	*P*=.498	χ^2^=14.524	***P*<.001**	χ^2^=16.956	***P*<.001**	χ^2^=0.171	*P*=.679

Income per month										
<10000SR	226/273	82.8	207/277	74.7	184/272	67.6	168/276	60.9	134/269	49.8
≥10000SR	120/154	77.9	108/155	69.7	102/155	65.8	92/155	59.4	62/153	40.5
	χ^2^=1.514	*P*=.219	χ^2^=1.284	*P*=.257	χ^2^=0.151	*P*=.697	χ^2^=0.095	*P*=.758	χ^2^=3.358	*P*=.066

Bolding indicates statistically significant difference.

Lack of time was reported by more than two thirds (67.6%) of the study group as a barrier to healthy diet. Lack of time was significantly higher among the younger age group and was inversely associated with age and was significantly higher among those never married. Lack of resources was stated by 60.2% of the study group as a barrier to a healthy diet. Lack of resources was significantly higher among younger age groups and was significantly higher among those never married.

## DISCUSSION

In the present study none of the participants met the recommendations of the ACSM/CDC for daily activity.^11^ Previous surveys estimated that physical inactivity prevalence ranged from 43.3% to 99%.^5^,^12^ A large population-based cross-sectional study published recently in Saudi Arabia showed a prevalence of only 3.9% for regular physical activity.^6^ WHO indicated that 60% to 85% of adults around the world are not active enough to achieve the benefits of physical activity.^2^ Physical inactivity in the Saudi population seems to be among the highest in the world. It is this high prevalence of inactivity and the high calorie diet intake that has lead to the epidemic of overweight and obesity, diabetes mellitus type 2, and coronary heart disease in recent years.^2^

Females were found to be much less active than males. This is consistent with other studies.^6^,^13^–^15^ However, one study found that Saudi women were moderately more active than men.^16^ This was explained by the fact that the former study assessed all types of physical activity, including house work while in our study only sport and leisure time physical activity were included. The main barriers to adherence to physical activity demonstrated in our study were lack of resources, lack of willpower, lack of social support and lack of energy. Lack of resources was the most common barrier reported, especially by females. This was not surprising as in Saudi Arabia there is limited access for women to join sport clubs, jogging trails, swimming pools or exercise facilities at work. Similar findings were found by other studies.^17^,^18^

Also, we showed that participants with income less than 10 000 SR/month believe that lack of resources is a barrier because it is expensive for them to have a class or join a club or buy the right equipment. One study reported that 52% of barriers to exercise were due to unavailability of affordable exercise venues.^17^ Indoor walking trails and affordable exercise venues should be encouraged in Saudi Arabia due to the hot weather which extends for about 6 months and which affects walking during that period.

Lack of willpower ranked as the second barrier to adherence to regular physical activity. It is easier to find excuses not to exercise than to go out and do something. Lack of will power is a result of lack of self motivation. The main motivators reported for exercising in the younger age group were fitness and fun; for the older age group it was good health.^18^ Family physicians were reported to be the most important source of support for adopting a healthy lifestyle.^8^,^19^

Other exercise barriers found in this study were lack of social influence and lack of energy, both mostly reported by females. Support of friends and joining groups to provide companionship while being physically active is strongly needed. There is strong evidence that social support increased time spent in activity by approximately 44%. However, there is insufficient evidence for the effectiveness of family-based social support.^11^ Lack of energy was reported by the CDC to be among the top three barriers to exercise.^8^,^20^ However, in our study it ranked as the fourth. Reasons could be explained by the fact that 30% of the participants are married housewives. Working women are more likely to be exposed to stress and exhaustion and thus to lack of energy compared to housewives. One study showed that women reported stress as a barrier to healthy lifestyle significantly more often than men.^19^

In the present study lack of time was not considered a barrier to exercise, which is contrary to what has been published.^8^,^18^,^21^ Reasons could be related to the fact that 68% of the participants were females and half of them were housewives. Furthermore, a large number of families employ housemaids in their homes, with the result that there is more free time for women.

Diet is a powerful instrument in preventing NCDs. In Finland, the North Karelia project, through community-based activity encouraged a healthier diet that resulted in annual coronary heart disease mortality reduction of 73% over 25 years.^22^ In the present study barriers to a healthy diet were lack of willpower followed by lack of social support and lack of time and resources. Lack of willpower was the commonest barrier for a healthy diet. It was also mostly reported by married and middle-aged participants. It seems that it is difficult to give up favorite foods and substitute with healthy foods, especially if the individual is living with a family. This is similar to the results of studies in Kuwait and Spain where willpower was among the main barriers to adherence to a healthy diet.^21^,^23^

Lack of social influence was mostly reported by participants of middle age and of less than university education. It is clear that lifestyle change interventions are more effective when others close to the individuals are involved.^11^ Family, friends, neighbors, and co-workers represent potential resources for maintaining lifestyle changes. Also, large numbers of social gatherings with extended families interfere with adherence to a healthy diet.^21^ Lack of time and resources were barriers for healthy diet among younger and never married individuals. Busy lifestyles and a paucity of restaurants with healthy food choices have led the younger age group to consume fast food. Similarly, a study conducted among European countries found that lack of time was the most frequently (24%) mentioned barrier for adherence to healthy diet.^24^

In conclusion, the lack of resources was the most important barrier for physical activity, while lack of willpower and social support were both barriers for adherence to physical activity and healthy diet. Improvements in the physical environment and infrastructure are needed with more access to affordable healthy food choices. Also, a multi-sectored approach is required to increase the awareness of the health benefits of physical activity and healthy diet and to promote behavior change at the individual level. Further research is needed by means of a national survey to compare barriers to healthy lifestyle among different regions of Saudi Arabia. This will help to tailor strategies according to the barriers detected. The limitation of this study was that most of the participants were mainly governmental employees. It did not include those who receive private or military hospital services. Thus, the results cannot be generalized to the whole population.

**Figure 1:**
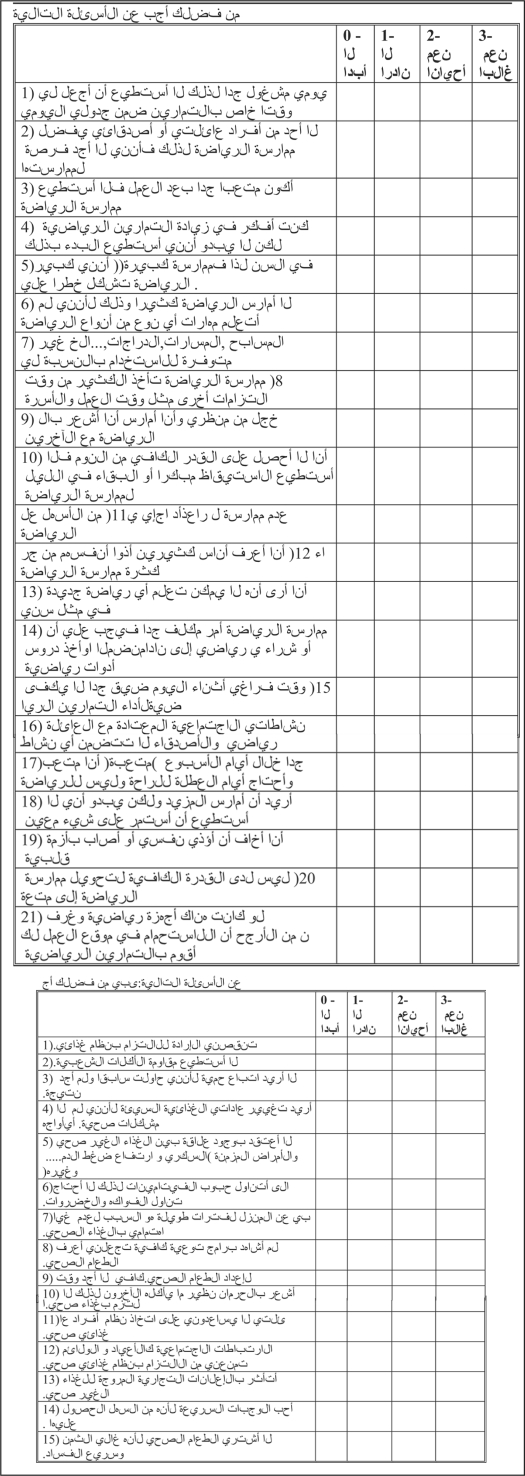
Arabic questionnaire.
